# Time to restore body weight in adults and adolescents receiving cognitive behaviour therapy for anorexia nervosa

**DOI:** 10.1186/s40337-015-0057-z

**Published:** 2015-05-28

**Authors:** Simona Calugi, Riccardo Dalle Grave, Massimiliano Sartirana, Christopher G Fairburn

**Affiliations:** Department of Eating and Weight Disorder, Villa Garda Hospital, Via Montebaldo, 89, I-37016 Garda, VR Italy; Department of Psychiatry, Oxford University, Warneford Hospital, Oxford, OX3 7JX UK

**Keywords:** Anorexia nervosa, Treatment, Cognitive behaviour therapy, Eating disorder, Outcome

## Abstract

**Background:**

The aim of the present study was to provide benchmark data on the duration of treatment required to restore body weight (to BMI ≥18.5 or a corresponding BMI centile) in adolescents and adults with anorexia nervosa treated with outpatient cognitive behaviour therapy.

**Methods:**

Ninety-five participants (46 adolescents and 49 adults) were recruited from consecutive referrals to a specialist eating disorder clinic. Each was offered 40 sessions of enhanced cognitive behaviour therapy (CBT-E) over 40 weeks, the conventional length of this treatment.

**Results:**

Twenty-nine (63.1%) of the adolescents and 32 (65.3%) of the adults completed all 40 sessions of treatment (P = 0.818). Significantly more adolescents reached the goal BMI than adults (65.3% vs. 36.5%; P = 0.003). The mean time required by the adolescents to restore body weight was about 15 weeks less than that for the adults (14.8 (SE = 1.7) weeks vs. 28.3 (SE = 2.0) weeks, log-rank = 21.5, P < 0.001).

**Conclusions:**

The findings indicate that adolescent patients receiving CBT-E are able to regain weight more successfully than adults and at a faster rate. If these findings are replicated and extend to eating disorder psychopathology, then their treatment could be shorter than that of adults.

## Background

Anorexia nervosa is considered one of the most difficult psychiatric disorders to treat [1]. Patients are often reluctant to accept treatment and among those who start a large subgroup has a poor outcome. Anorexia nervosa is also difficult to study [2-6] because is relatively rare, associated with medical risks, and may require a lengthy duration of treatment. These difficulties have led to the recommendation that new treatments should be extensively tested prior to being evaluated in randomized controlled trials [2-6].

Enhanced cognitive behaviour therapy (CBT-E) is a treatment for eating disorder psychopathology, irrespective of the eating disorder diagnosis [7-9]. There are data from randomized control trials supporting its use in bulimia nervosa, and the other non-underweight eating disorder presentations [10-12], and from randomized and observational studies of adolescents and adults with anorexia nervosa [13-17]. When used to treat patients who are underweight CBT-E generally involves about 40 sessions over 40 weeks [8] (i.e., about twice the length of CBT-E for patients who do not need to regain weight). This length of treatment is mainly to accommodate the time needed for weight regain. The optimal duration of treatment has not been established, and it may be shorter for adolescents than adults as it has been suggested that they are easier to treat [5].

## Purpose

The aim of the present study was to obtain benchmark data on the duration of treatment required to restore body weight in adolescents and adults with anorexia nervosa treated with outpatient CBT-E.

## Methods

### Design

A cohort of participants with anorexia nervosa was recruited from consecutive referrals to an eating disorder clinic. Eligible participants were offered 40 sessions of CBT-E over 40 weeks. This was the sole psychological intervention that they received. The study was approved by the local human subjects committee.

### Setting and participants

The sample was recruited from consecutive referrals by family doctors and other clinicians to a well-established eating disorder clinic serving the Verona area of Italy. The patients were required to fulfill DSM-IV diagnostic criteria for anorexia nervosa [18] bar the amenorrhea criterion. In addition, for patients under 18 years, the patient’s parents or legal guardians had to provide written informed consent to their participation after having received a full description of the study.

One hundred and fifty-two patients were screened, of whom 32 did not meet the inclusion criteria. Fifteen met the following exclusion criteria: (i) extremely underweight (BMI < 14) (N=1); (ii) marked medical complications (e.g., pronounced edema, severe electrolyte disturbance, hypoglycemia) (N = 1); (iii) having received in the previous year a specialist treatment for anorexia nervosa (N = 1); (iv) having a co-existing Axis 1 psychiatric disorder that precluded immediate eating disorder-focused treatment (e.g., psychosis or drug dependence, N = 6); and (v) not being available for the 40 week period of treatment (N = 6).

The remaining 105 patients were offered CBT-E, of whom 95 accepted (90.5%). A detailed description of the participants has been provided elsewhere [13,14].

### The treatment

CBT-E is a treatment for people with eating disorder psychopathology, irrespective of their eating disorder diagnosis. A detailed description of the treatment has been published [8]. In brief, CBT-E for patients who are underweight has three main steps. The goal of the first step is to help patients see the need for weight regain and decide to embark upon it. The goal of the second step is to help patients regain weight to a low-healthy level, and at the same time address their eating disorder psychopathology. In adults the goal BMI is between 19.0 and 19.9. In adolescents it is the equivalent BMI centile. In the third step, the goal is to help the patients maintain their new healthy weight. In the present study, CBT-E involved 40 one-to-one treatment sessions over 40 weeks. The 40 sessions were preceded by two preparatory sessions, and followed by a review session 20 weeks after completing treatment. The patients were considered “completers” if they attended to all the 40 sessions of treatment.

CBT-E for adults and adolescents is essentially the same. It differs in just one way. In adults significant others (friends, partner or parents) are only seen if it is thought likely that it will be beneficial and with the consent of the patient. This only applies in a minority of cases. When it happens, the role of the significant other is simply to support the implementation of the one-to-one treatment. The same principles apply to the treatment of adolescent patients except that the parents are invariably involved given these patients’ age and circumstances. In the present study, the great majority of sessions involved the adolescent patient alone. Parental involvement comprised a single one-hour assessment session in the first two weeks of treatment and eight 15-minute sessions with the patient and parents together (immediately after an individual session). These occurred at weeks 1 to 4 and at weeks 8, 12, 20, and 40. The aim of the initial session with parents was to identify family factors liable to hinder the patient’s attempts to change while the subsequent sessions were devoted to meal planning, the conduct of mealtimes and to the generation of solutions to problems that had emerged or were foreseeable. Additional sessions with the parents only took place if there were family crises, extreme difficulties at mealtimes or parental hostility towards the adolescent. Few such sessions were needed.

A single therapist treated each patient. There was no additional therapeutic input, either from physicians, dieticians or other health professionals, unless there was a specific indication (e.g., the management of medical complications or comorbid conditions). The sole additional input was assessment by a physician (Riccardo Dalle Grave, RDG) prior to starting treatment and, rarely, a reassessment if there were medical concerns (e.g., weight loss of over 0.5 kg in a week).

There were four therapists, all of whom were clinical psychologists. All the therapists had prior generic clinical experience and experience in treating patients with eating disorders, and each received six months initial training from Christopher G Fairburn (CGF) and RDG. Weekly supervision meetings were held throughout the study and were led by RDG. The therapists had six-monthly booster workshops led by CGF. All the treatment sessions were recorded and these recordings were used as part of supervision to ensure that the treatment was well implemented.

### Assessment and measures

#### Body weight and body mass index

Weight was measured using a beam balance scale and height was measured using a wall-mounted stadiometer. Participants were weighed wearing only underwear and without shoes. The body mass index (BMI) was determined for adults according to the standard formula of body weight measured in kilograms divided by height in meters squared. BMI centiles were calculated for adolescents using the Center for Disease Control and Prevention growth charts (www.cdc.gov/growthcharts). BMI and BMI centiles were measured each week throughout the duration of the treatment.

#### Eating disorder features and general psychopathology

The Italian version of the self-report Eating Disorder Examination Questionnaire (EDE-Q6.0) was used to measure eating disorder psychopathology. It was administered at baseline and at the end of treatment [19,20].

The full version of the Symptom Checklist-90 (SCL-90) was used to assess general psychopathology from which a Global Severity Index (GSI) was calculated [21,22]. The GSI was measured at baseline and at the end of treatment.

The Italian language version of the questionnaires has good psychometric properties [20,22].

### Statistical analysis

The demographic and baseline clinical characteristics of the adolescent and adult patients were compared using t-test, Mann–Whitney test or chi-square test, as appropriate.

A Kaplan-Meier estimate was used to evaluate differences on time to restore body weight, in the adult and adolescent patients respectively, during the 40 weeks of treatment. Two BMI thresholds were investigated; reaching a BMI ≥18.5 and reaching a BMI ≥19.5, or in the adolescents achieving the corresponding BMI centile [23]. A Cox regression model was used to analyze the independent baseline predictors (age, BMI, duration of illness, EDE-Q total score, SCL-90 GSI, number of binge eating and purging episodes) on time to reach BMI ≥ 18.5 or corresponding BMI centile. Data on patients who did not reach these values were censored at 40 weeks. Dropouts for clinical reasons or for being admitted to inpatient treatment were censored at the date of interruption of the outpatient treatment. The proportional hazards assumption of the Cox regression model was tested using the log-log plot. With this method, a plot of the logarithm of the negative logarithm of the estimated survivor function against the logarithm of survival time yields parallel curves if the hazards are proportional across the adolescent and adult groups [24]. The log-log plot showed that the two curves looked approximately parallel, indicating that the proportional hazards assumption was met.

## Results

### The sample

The sample comprised 95 patients, 46 adolescents and 49 adults. Table 1 shows their baseline characteristics. As would be expected, the adults had a longer duration of disorder than the adolescents and a greater proportion reported binge eating, self-induced vomiting, and laxative misuse.

**Table 1:** **Baseline clinical characteristics in adolescent and adult patients with anorexia nervosa**

	Adolescents (N = 46)	Adults (N = 49)	Tests
**Age** **, years**	15.5 (1.3)	24.6 (5.2)	t = 11.78***
**Gender**, **n (%) female**	46 (100%)	48 (98.0%)	χ^2^ = 0.95
**Marital status**, **n (%)**			χ^2^ = 4.95
- single, never married	46 (100%)	42 (89.4%)	
- married or living as such	0	2 (4.3%)	
- separated or divorced	0	3 (6.4%)	
**Occupation** **, n (%)**			χ^2^ = 35.41***
- full-time employment	0	20 (40.1%)	
- student	46 (100%)	22 (44.9%)	
- homeworker	0	5 (10.2%)	
- unemployed	0	2 (4.1%)	
**Duration of eating disorder,** **years, median (range)**	0.5 (0–5)	3.0 (0–17.0)	Z = 4.17***
**Weight**			
- body weight (kg)	40.0 (5.7)	41.6 (6.0)	t = 1.33
- body mass index (kg/m^2^)	--	15.7 (1.4)	
- body mass index centile^	2.86 (3.35)	--	
- weight 95% of that expected, n (%)	1 (2.2)	--	
**Eating disorder psychopathology**			
- overall severity (global EDE-Q)	2.79 (1.5)	2.87 (1.5)	t = 0.26
- global EDE-Q <1SD above the community mean^#^, n(%)	18 (41.9%)	22 (46.8%)	χ^2^ = 0.32
- dietary restraint (EDE-Q subscale)	2.69 (1.8)	3.08 (2.0)	t = 1.00
- eating concern (EDE-Q subscale)	2.58 (1.5)	2.68 (1.6)	t = 0.31
- shape concern (EDE-Q subscale)	3.17 (1.7)	3.00 (1.7)	t = 0.49
- weight concern (EDE-Q subscale)	2.75 (1.6)	2.69 (1.5)	t = 0.19
**Eating disorder behaviour (EDE-Q)**			
- objective binge eating, n(%) present	2 (4.3%)	12 (24.5%)	χ^2^ = 7.66**
if present, episodes/28 days, median(range)	38.5 (7–70)	4.0 (1–40)	
- self-induced vomiting, n(%) present	1 (2.2%)	16 (32.7%)	χ^2^ = 15.00***
if present, episodes/28 days, median(range)	120	9.5 (1–60)	
- laxative misuse, n(%) present	1 (2.2%)	8 (16.3%)	χ^2^ = 5.54*
if present, episodes/28 days, median(range)	12	11.5 (3–84)	
**General psychiatric features, GSI**	1.18 (0.6)	1.36 (0.7)	t = 1.34

#Global EDE-Q less than 1SD above community EDE-Q mean for young adult women [25] (i.e., <2.77).

^ BMI centile as 0.5 for those with a value < 1.

EDE-Q – Eating Disorder Examination Questionnaire (version 6.0).

GSI – SCL-90 Global Severity Index.

*p < 0.05; **p < 0.01; ***p < 0.001 adolescent vs. adult patients.

Data are shown as mean (SD) unless otherwise indicated.

#### Treatment completion

Sixty-one participants (64.2%) completed the full 40 weeks of treatment (completers). The remainder (non-completers) either left the program prematurely or were referred for more intensive treatment. No significant differences were found between the completers and non-completers on demographic (age, gender, marital status, occupation) and baseline clinical (duration of illness, weight, BMI, global EDE-Q, SCL-90 GSI) characteristics. The non-completion rate was similar in the adolescents and adults (36.9% vs. 34.7% respectively; χ^2^(1,94) = 0.20, P = 0.839). Eating disorder and general psychopathology significantly improved, as described in the two previous papers [14,13], with there being no differences between the adults and adolescents at the end of treatment (mean global EDE-Q: 1.8 (SD = 1.7) vs 1.5 (SD = 1.5), respectively P = 0.365; mean SCL-90 GSI: 0.9 (SD = 0.7) vs 0.7 (SD = 0.6), respectively P = 0.139).

#### Weight regain

**Figure 1:**
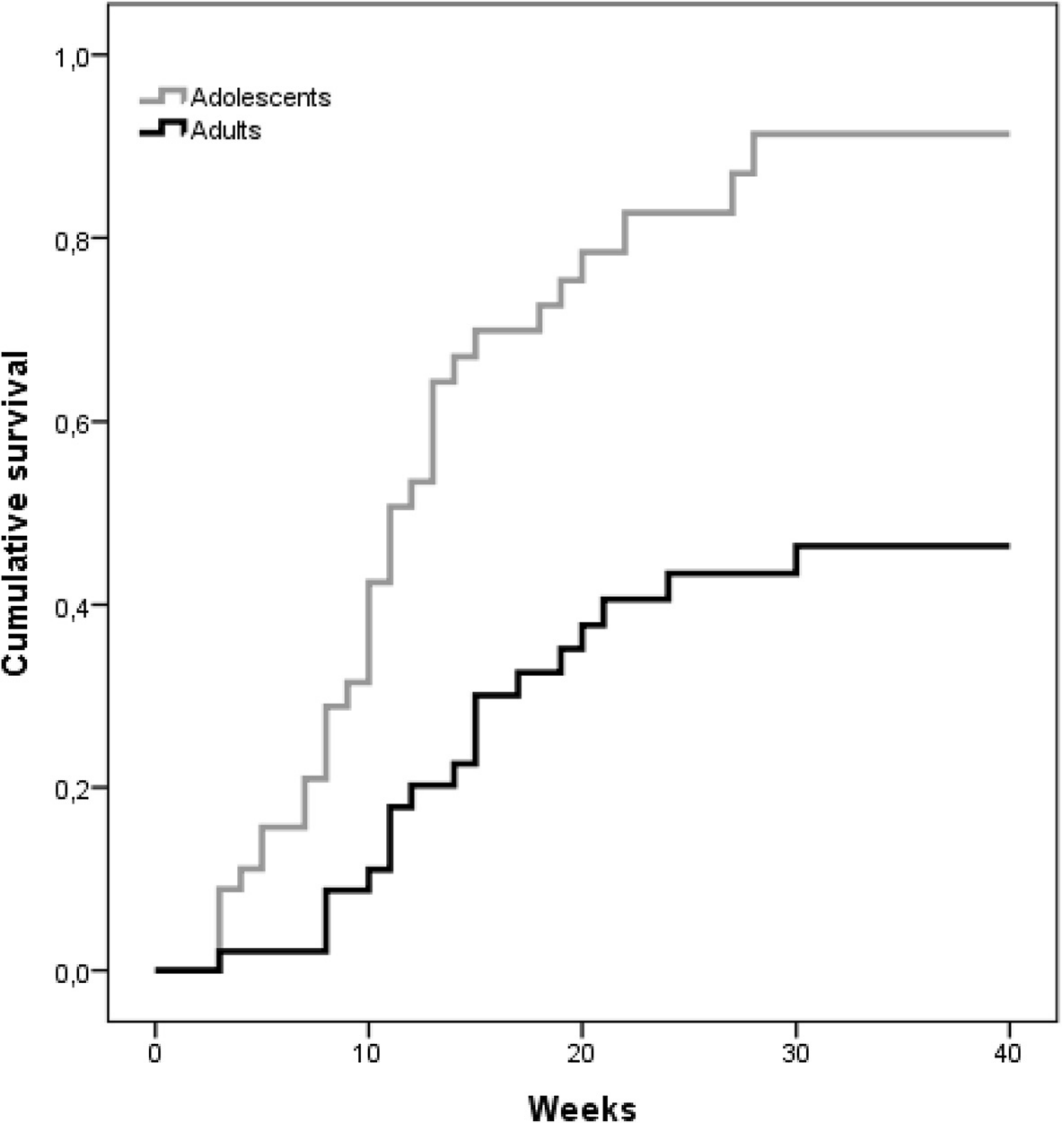
Survival curves predicting time to reach a BMI ≥ 18.5 (or corresponding BMI centile) in adult and adolescent patients with anorexia nervosa.

During the treatment, 32 of the adolescent patients (65.3%) had reached a BMI ≥18.5 or the corresponding centile compared with 19 of the adults (36.5%; χ^2^(1,94) = 9.05, P = 0.003). Among the adolescents and adults who reached their target BMI, no difference was found in the amount of weight regained (5.8 (SD = 3.7) vs 6.4 (SD = 3.0) kg, respectively; t = 0.61, P = 0.542); however, the estimated mean time needed to achieve this weight goal was about 15 weeks less for the adolescents than for the adults (14.8 (SE = 1.7) v. 28.3 (SE = 2.0) weeks, log rank = 21.5, P < 0.001) (Figure 1). Among the patients who reached a BMI ≥18.5 or the corresponding centile, 96.6% of the adolescents and 80.0% of the adults maintained this weight at the end of treatment.

Similar results were found using a target BMI of 19.5 or more or the corresponding BMI centile [9]. Over half of the adolescents (55.1%) and a third of the adults (32.6%; χ^2^(1,94) = 3.95, P = 0.047) achieved this BMI, and the time taken was about 11 weeks less for the adolescents than the adults (25.1 (SE = 2.1) v. 36.4 (SE = 1.4) weeks, log rank = 23.0, P < 0.001).

A Cox regression model including demographic and baseline clinical characteristics showed that age and baseline BMI were the only two independent predictors of time required to reach a BMI ≥18.5 or corresponding BMI centile (Wald Chi-Square = 4.06, df = 1, P = 0.044, OR = 0.91, 95% CI 0.82-0.99; Wald Chi-Square = 13.35, df = 1, P < 0.001, OR = 1.85, 95% CI 1.33-2.58, respectively). Age was the only independent predictor of time required to reach a BMI ≥19.5 or corresponding BMI centile (Wald Chi-Square = 10.29, df = 1, P = 0.001, OR = 0.80, 95% CI 0.70-0.92).

## Discussion

The aim of the study was to obtain benchmark data on the duration of treatment required to restore body weight in adolescents and adults with anorexia nervosa treated with outpatient CBT-E. There were two main findings. The first was that more adolescents than adults reached the two target BMIs. The second was that the adolescents did so at a faster rate: for example, the time taken to reach a BMI centile equivalent to 18,5 was about 15 weeks less than that for the adults. The time to reach the BMI ≥18.5 and ≥19.5 or the corresponding centile was associated with younger age and higher baseline BMI and younger age, respectively.

The present study had certain strengths. First, the two samples were large for studies of the treatment of anorexia nervosa; second, the patients were recruited from consecutive referrals to a long-established eating disorder clinic that provides the main clinical service for the local area. With regard to the baseline characteristics, our adult sample had a similar BMI to patients with anorexia nervosa described by the CBT outpatient study of Turner et al. [26], and a shorter average duration of eating disorder of patients with anorexia nervosa described by the CBT-E study of Byrne et al. [27]. No CBT or CBT-E studies on adolescents with anorexia nervosa are available for comparison. Our findings are therefore likely to be generalizable to clinical services elsewhere.

Third, and in contrast with most studies of the treatment of anorexia nervosa, the index treatment, CBT-E, was the patients’ sole treatment with there being no other accompanying interventions. Lastly, the form of CBT-E used was essentially the same for the adolescents and adults. It is therefore likely that differences observed between the two age groups were genuine rather than being due to differences between the forms of CBT-E employed.

The study also had some limitations. First, the treatment was delivered in a single outpatient unit. Replication is therefore needed. Second, we focused on BMI as the target variable rather than eating disorder psychopathology. We chose BMI as it is a key outcome variable in the treatment of anorexia nervosa and achieving a healthy BMI is one of the main determinants of length of treatment. A similar analysis focused on remission of eating disorder psychopathology would be of interest.

## Conclusions

The findings indicate that adolescent patients receiving CBT-E are able to regain weight more successfully than adults and at a faster rate. If these findings are replicated and extend to eating disorder psychopathology, then their treatment could be shorter than that of adults.
